# Bullous Pemphigoid Following the Moderna mRNA-1273 Vaccine

**DOI:** 10.7759/cureus.24126

**Published:** 2022-04-13

**Authors:** Amar D Desai, Radhika Shah, Attiya Haroon, Cindy Wassef

**Affiliations:** 1 Department of Dermatology, Rutgers University New Jersey Medical School, Newark, USA; 2 Dermatology, Rutgers Robert Wood Johnson Medical School, New Brunswick, USA

**Keywords:** scars, bullous pemphigoid, vaccination reaction, moderna mrna 1273, moderna vaccine, sars-cov-2 vaccine, covid-19 vaccine

## Abstract

As the onset of novel variants of the severe acute respiratory syndrome coronavirus 2 virus pushes policy-makers to push widespread vaccination efforts, it is likely that an increased number of severe cutaneous adverse reactions (SCARs) will present. Therefore, it is important to understand the presentation of possible SCARs. However, data are limited regarding which SCARs are most likely to be found following vaccination, and specific presentations in certain demographic groups, such as postmenopausal women, remain widely unknown. Here, we present the case of a 73-year-old female with no medical history or allergies presenting with a unique reaction of systemic bullous pemphigoid following the Moderna mRNA-1273 vaccine. To our knowledge at the time of this writing, based on a thorough review of the literature using PubMed, no such cases exist following the Moderna vaccine in the United States in elderly, postmenopausal women. We present a brief discussion on the presentation and management to hopefully alleviate future morbidity from similar reactions with increased distribution of the vaccine.

## Introduction

Following the global spread of the severe acute respiratory syndrome coronavirus 2 (SARS-CoV-2) virus, also known as coronavirus disease 2019 (COVID-19), in late 2019 and early 2020, it has claimed over 984,000 deaths in the United States with millions more infected [1]. The COVID-19 vaccine rollout has proven to be an effective source in diminishing the transmission of the virus nationally, with multiple clinical trials proving the safety and efficacy of these vaccines [2]. However, numerous extremely rare side effects from the vaccine have been reported, including anaphylaxis, thrombosis (following the Janssen Ad26.COV2.S vaccine), myocarditis, and Guillain-Barré syndrome [3,4]. Previous cutaneous adverse effects of the vaccine include cases of erythematous plaques within several days of receiving the vaccine, pernio, zoster, herpes simplex flares, and pityriasis-related reactions [5,6]. Given that many published studies on these adverse effects have also been in the adult population due to the age restrictions of the vaccine, it is also possible that different and possibly more severe cutaneous adverse effects will be observed in children during further rollout [7,8].

As the onset of the omicron variant, and further spread of the delta variant, of the SARS-CoV-2 pushes policy-makers to push widespread vaccination efforts, it is likely that an increased number of severe cutaneous adverse reactions (SCARs) will present. Here, we report a case of bullous pemphigoid which developed following the Moderna mRNA-1273 vaccine.

## Case presentation

**Figure 1:**
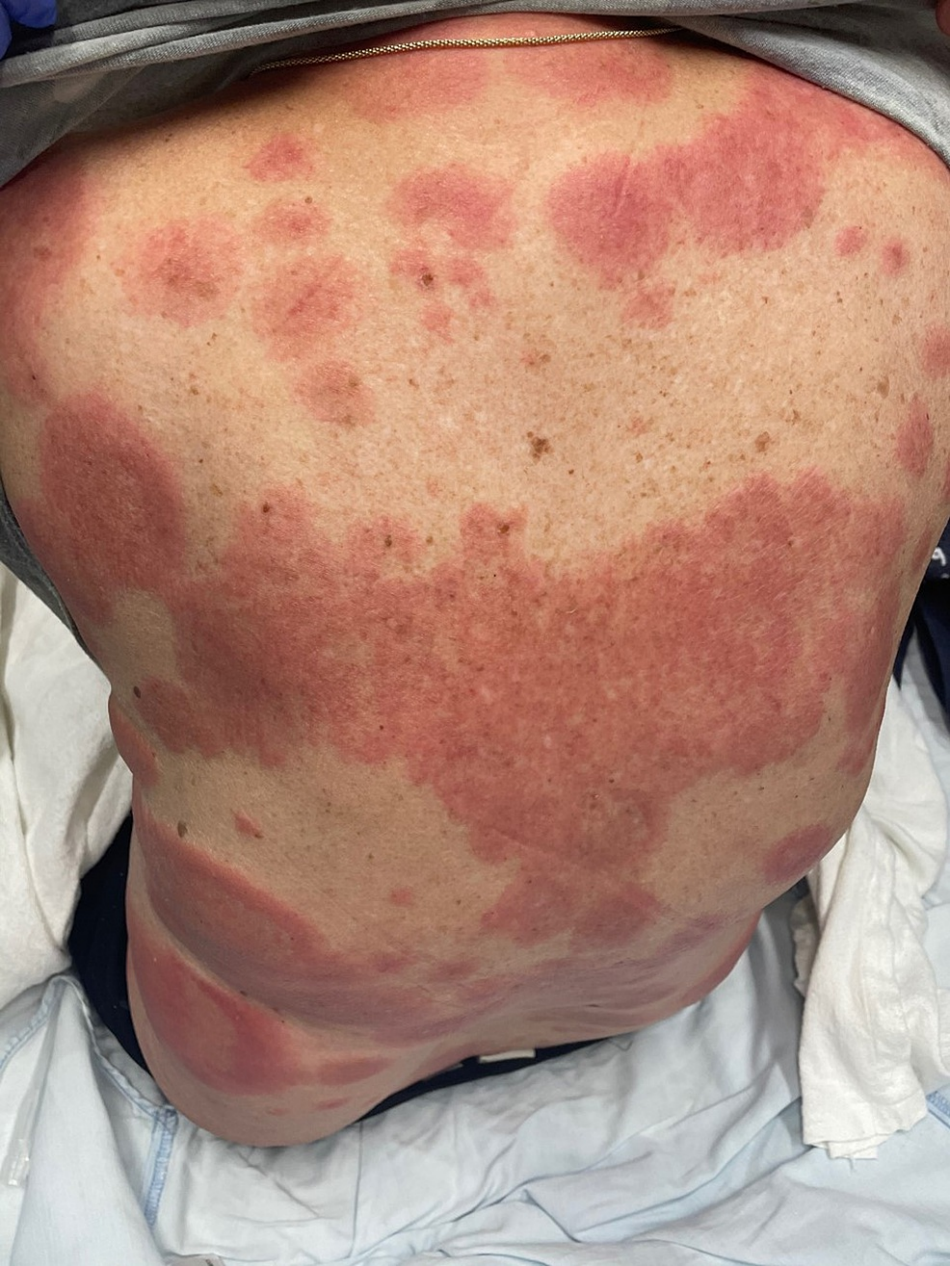
Skin lesions on the patient’s back.

**Figure 2:**
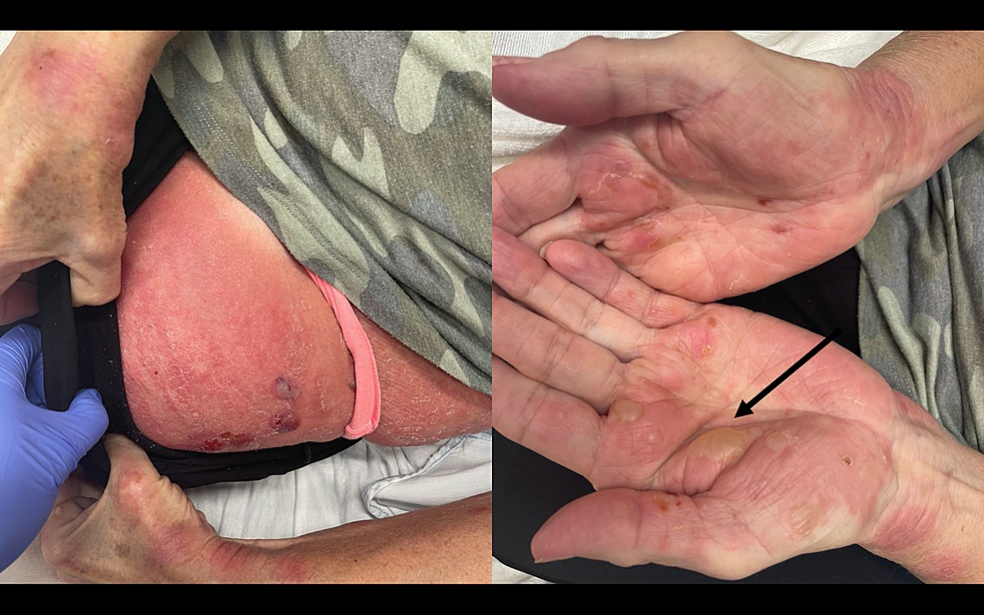
Lesions on the patient’s thigh (left) and hands (right).

A 73-year-old female with no medical history presented to the emergency department with a new-onset generalized collection of intensely pruritic eruptions which first arose 24 hours after receiving the second dose of the Moderna mRNA-1273 vaccine. It started on the trunk and later spread to the extremities and face, sparing the genital and oral mucosa. On physical examination, tense, filled bullae were noted throughout her trunk (Figure 1), extremities (Figure 2), and face, ranging in size from 5 mm to 2 cm. The patient denied fever, joint pain, and weight loss associated with the episode. She also denied prior allergies, recent illnesses, or a family history of dermatologic or autoimmune conditions. Of note, the patient also reported a similar eruption within 24 hours following the first dose of her vaccine. She received two short (seven-day) courses of oral corticosteroids that would improve the rash but they would return upon completion of the steroid course.

A biopsy was subsequently performed on the patient, with a sample being taken from a bulla on the left upper thigh, revealing a subepidermal blistering disorder with numerous eosinophils. A direct immunofluorescence test was performed from the same site as the biopsy, revealing a linear deposition pattern of C3 and immunoglobulin G (IgG) at the dermal-epidermal junction consistent with bullous pemphigoid. A diagnosis of bullous pemphigoid was made and the patient was subsequently started on prednisone 1 mg/kg and mycophenolate mofetil steroid-sparing agent. After a week of treatment, the patient had improvement in her symptoms.

## Discussion

SCARs from the administration of COVID-19 vaccines have been documented before; however, these mostly consist of variations of drug-induced hypersensitivity reactions, and, less commonly, DRESS syndrome or Stevens-Johnson syndrome [6]. The timing of these SCARs seems to match that seen in this specific patient, with high susceptibility for initial onset of a reaction within the first 24 hours following administration. However, new-onset cutaneous symptoms have been reported even several days after administration. Previous case reports have shown bullous pemphigoid arising after vaccination for diphtheria, tetanus, pertussis, poliomyelitis, hepatitis B, and *Hemophilus influenzae B*; bullous pemphigoid has also been reported after infection with viral herpetic stomatitis [9]. Most of these reactions occurred over 24 hours of the initial vaccine administration, most commonly following a two-day period. Notably, several of these vaccine reactions were found in the pediatric population. Due to the more recent rollout of the COVID-19 vaccines in the pediatric population after being deemed safe [10], the possibility of an increased incidence of bullous pemphigoid as a possible cutaneous reaction should be considered during the vaccine rollout in this younger demographic [11]. Therefore, while the exact triggers for the pathogenesis of bullous pemphigoid remain unknown, it has been previously shown to erupt days after both vaccinations and infections, suggesting that the humoral pathway which is responsible for the eruption of similar non-vaccine-related pemphigoid reactions is likely at play. The risk of autoimmune disease flare-ups after COVID-19 vaccines has been previously suggested but appears to be rare in nature, moderate in severity, and typically responsive to treatment [12,13].

These isolated cases suggest that, like many other vaccines and infections, the COVID-19 vaccine may present no differently in serving as a possible immune trigger for a flare-up of autoimmune conditions such as bullous pemphigoid. As vaccine trials expand, the inclusion of patients with pre-existing autoimmune conditions, as well as the collection of information regarding features of previously healthy patients who develop autoimmune disease or flare-ups following vaccination, will be important in better understanding the risks and management of these individuals. As shown in this case, these flare-ups are likely responsive to treatment and preventable with possible precautionary measures such as prior screening for higher-risk individuals with certain human leukocyte antigen (HLA) types and autoimmune conditions and inclusion of them in vaccine trials. While definitive associations between certain HLA types and reactions to these vaccines do not exist yet, further research may enable this type of early detection. Protection of these communities from SARS-CoV-2 remains an important consideration, especially because of the onset of novel variants and increased incidence.

## Conclusions

Here, we present a case of a 73-year-old female with no medical history who presented with a new-onset generalized bullous pemphigoid reaction following the second dose of the Moderna mRNA-1273 vaccine. We suggest that the COVID-19 vaccine, much like many other vaccines, may serve as a trigger for autoimmune reactions such as bullous pemphigoid even in patients with no significant medical history. Furthermore, these SCARs may not flare up immediately, underscoring the importance of communication with patients regarding possible future side effects which may evolve and steps to seek care if they do. Lastly, given a growing number of variants, including the novel omicron and delta variants which have spread throughout the United States, it is important that future research, especially in the pediatric population yet to be exposed to these vaccines, is performed to assess the best management of these SCARs as vaccine rollout continues.
